# Tumor-Shed PGE_2_ Impairs IL2Rγc-Signaling to Inhibit CD4^+^ T Cell Survival: Regulation by Theaflavins

**DOI:** 10.1371/journal.pone.0007382

**Published:** 2009-10-08

**Authors:** Sreya Chattopadhyay, Sankar Bhattacharyya, Baisakhi Saha, Juni Chakraborty, Suchismita Mohanty, Dewan Md. Sakib Hossain, Shuvomoy Banerjee, Kaushik Das, Gaurisankar Sa, Tanya Das

**Affiliations:** Division of Molecular Medicine, Bose Institute, P-1/12 CIT Scheme VIIM, Kolkata, India; Bauer Research Foundation, United States of America

## Abstract

**Background:**

Many tumors are associated with decreased cellular immunity and elevated levels of prostaglandin E2 (PGE2), a known inhibitor of CD4+ T cell activation and inducer of type-2 cytokine bias. However, the role of this immunomodulator in the survival of T helper cells remained unclear. Since CD4+ T cells play critical roles in cell-mediated immunity, detail knowledge of the effect tumor-derived PGE2 might have on CD4+ T cell survival and the underlying mechanism may, therefore, help to overcome the overall immune deviation in cancer.

**Methodology/Principal Findings:**

By culturing purified human peripheral CD4+ T cells or Jurkat cells with spent media of theaflavin- or celecoxib-pre-treated MCF-7 cells, we show that tumor-shed PGE2 severely impairs interleukin 2 receptor γc (IL2Rγc)-mediated survival signaling in CD4+ T cells. Indeed, tumor-shed PGE2 down-regulates IL2Rγc expression, reduces phosphorylation as well as activation of Janus kinase 3 (Jak-3)/signal transducer and activator of transcription 5 (Stat-5) and decreases Bcl-2/Bax ratio thereby leading to activation of intrinsic apoptotic pathway. Constitutively active Stat-5A (Stat-5A1*6) over-expression efficiently elevates Bcl-2 levels in CD4+ T cells and protects them from tumor-induced death while dominant-negative Stat-5A over-expression fails to do so, indicating the importance of Stat-5A-signaling in CD4+ T cell survival. Further support towards the involvement of PGE2 comes from the results that (a) purified synthetic PGE2 induces CD4+ T cell apoptosis, and (b) when knocked out by small interfering RNA, cyclooxygenase-2 (Cox-2)-defective tumor cells fail to initiate death. Interestingly, the entire phenomena could be reverted back by theaflavins that restore cytokine-dependent IL2Rγc/Jak-3/Stat-5A signaling in CD4+ T cells thereby protecting them from tumor-shed PGE2-induced apoptosis.

**Conclusions/Significance:**

These data strongly suggest that tumor-shed PGE2 is an important factor leading to CD4+ T cell apoptosis during cancer and raise the possibility that theaflavins may have the potential as an effective immunorestorer in cancer-bearer.

## Introduction

Prostaglandins are lipid molecules regulating numerous processes including modulation of immune function [1]–[3]. PGE_2_ is produced by many different cell types, including malignant cells, and is known to contribute to cellular immune suppression in cancer patients [4], [5]. On the other hand, deletion of the respective prostaglandin receptors leads to reduced carcinogenesis and enhanced antitumor immunity [6].

Current paradigms suggest that CD4^+^ T cells play critical roles in the optimal induction and maintenance of clinically beneficial tumor immunity [7], [8]. These cells prime CTL-mediated antitumor responses [9] by preventing activation-induced cell death and functioning as antigen-presenting cells for CTLs to preferentially generate immune memory cells [10], [11]. CD4^+^ helper T cells also induce CD8^+^ cytotoxic T cell responses through dendritic cell activation by CD40/CD40L interactions [12] and determine the magnitude and persistence of such responses as well as CD8^+^ T cell infiltration of tumors [13]. Therefore, in order to establish itself, a growing tumor tries to overpower CD4^+^ T cells. It has been reported that both human patients and experimental animals with advanced cancer often exhibit a poorly functioning immune system [14]-[19]. There is evidence of increased apoptosis among CD4^+^ T cells in peripheral blood lymphocytes from cancer patients and animal models [20], [21]. Understanding the mechanisms of tumor-induced CD4^+^ T cell apoptosis as well as its amelioration by any biological response modifier is, therefore, of high importance from the point of view of amelioration of tumor-induced immuno-suppression.

Chemnitz *et al*. [22] have reported impairment in CD4^+^ T cell activation in cancer patients by PGE_2_. Tumor-shed PGE_2_ has been found to mediate profound alteration in cytokine balance in the cancer microenvironment and thereby contributing to T cell suppression in cancer patients [23], [24]. In fact, IL2 and IL2Rγc gene expression, that play crucial role in T cell proliferation, survival, and programmed cell death [25], are both targets of PGE_2_-induced suppression [26], [27], possibly through the inhibition of early events in T-cell signaling that include calcium influx and phosphatidylinositol breakdown [28], [29]. Moreover, PGE_2_ has been reported to down-regulate Jak-3 protein, which associates with IL2R, in T cells [26], [27]. This reduction in Jak-3 resulted in impaired phosphorylation and DNA binding activity of Stat-5 [26], [27]. Because Jak-3 is critical to IL2-dependent signaling and proliferation, its sensitivity to PGE_2_ may make it a prime target for suppressing IL2-dependent cell cycle progression in T cells. It is well accepted that Jak-mediated survival signals modulate Bcl-2 family of anti-apoptotic proteins [30]. Studies with Jak-3 deficient mice have showed down-regulation of Bcl-2 in CD8^+^ T cell population in thymus [31]. Other reports have demonstrated correlation between loss in Bcl-2 expression and death of T cells [32].

Interestingly, although PGE_2_ has been implicated in the increase or the acceleration of the programmed cell death process of immature CD4^+^CD8^+^CD3^+^ thymocytes in culture [33], CD4^+^CD8^+^ thymocytes inside the thymus in mice [34], and favors Th2-like cytokine secretion profiles in murine and human CD4^+^ T cells [35], there is no detail report on its effect on CD4^+^ T cell survival, if any, and the underlying mechanism. Since these helper T cells play critical roles in the optimal induction and maintenance of clinically beneficial tumor immunity [7], [8], here we address the role tumor-derived PGE_2_ might have on CD4^+^ T cell survival. Using exclusively primary human CD4^+^ T cells we found a correlation between over-expression of PGE_2_ in tumor cells and tumor-induced CD4^+^ T cell apoptosis. Inhibitory effects of tumor-shed PGE_2_ were in fact dependent on impairment of IL2Rγc signaling that lead to down regulation of IL2Rγc, reduced phosphorylation of Jak-3 and Stat-5A and decreased expression of pro-survival protein Bcl-2. Additionally, we demonstrated release of cytochrome c from mitochondria to cytosol and activation of caspase cascade. It was interesting to note that theaflavins, the black tea polyphenols, protected IL2Rγc signaling in CD4^+^ T cells from tumor-secreted PGE_2_ insult by inhibiting Cox-2 expression and subsequent PGE_2_ release from tumor cells. That these effects occurred in the absence of tumor cell-T cell contact negated the possibility of tumor contact-dependent CD4^+^ T cell apoptosis and highlighted PGE_2_ as an important mediator of impaired cellular immunity in patients with cancer.

There are ample evidences demonstrating the biological impact of theaflavins. These bioactive flavonoids of black tea have been reported to induce cell growth inhibition and apoptosis in a variety of cancer cells [36]–[38] Theaflavins also exert a plethora of beneficial effects on the cardiovascular system [39] and play a role in decreased intestinal cholesterol absorption thereby being responsible for lowering blood-cholesterol [40]. Antioxidative properties of theaflavins are manifested by their ability to inhibit free radical generation, scavenge free radicals and down-regulate the activity of pro-oxidative enzymes [41]. They can also influence activation of transcription factors such as NFkappaB or AP-1 [41]. However, there was hardly any report suggesting the immunoprotective effect of theaflavins. Our findings signify that theaflavins can be a possible therapeutic agent with a strong immunomodulatory effect and therefore, in future can be used alone or in combination with tumoricidal drugs to treat patients with cancer.

## Results

### Cell-free tumor supernatant leads to CD4^+^ T cell depletion by inducing apoptosis

**Figure 1:**
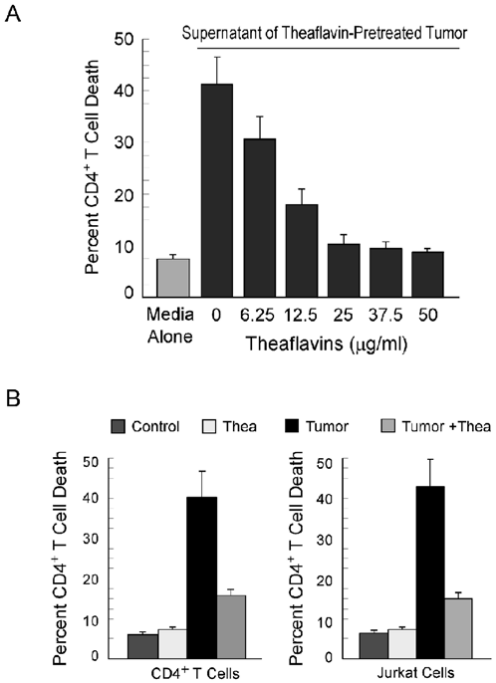
Cell free tumor supernatant leads to CD4^+^ T cell depletion by inducing apoptosis. A, Purified human peripheral CD4^+^ T cells were cultured in the presence of media alone or cell-free MCF-7-spent media (±theaflavins, doses from 6.25 µg/ml to 50 µg/ml). After 48 hours, viable cell numbers were scored by Trypan Blue exclusion method. B, Graphical representation of percent apoptosis of CD4^+^ T cells (*left panel*) and Jurkat T cells (*right panel*). CD4^+^ T cells labelled with Annexin V-PE and 7AAD were analyzed flow cytometrically. Annexin V/7AAD-positive cells were regarded as apoptotic cells. Values are mean±S.E.M. of five independent sets of experiments.

When purified CD4^+^ T cells were cultured in the presence of cell-free breast cancer cell (MCF-7) supernatants (Fig. 1A), a situation mimicking the tumor-bearing condition in which tumor-shed mediators influence the circulating CD4^+^ T cell repertoire, increase in the percent of dead CD4^+^ T cell (43.2%) in comparison to control (7.2%) was recorded. Interestingly, when these T cells were co-incubated with theaflavin-pretreated tumor culture supernatants, significant protection from tumor-induced death was observed in a theaflavin-dose-dependent manner, the optimum effect being at 25 µg/ml theaflavins (11.6% dead cells), beyond which no further significant change could be obtained (Fig. 1A). Subsequent studies were, therefore, carried out with this dose of theaflavins.

Depletion in CD4^+^ T cell populations by tumor supernatant prompted us to investigate the underlying cause. Next, to assess the mode of cell death, we used three-color flow cytometry (FITC-CD4, PE-Annexin-V and 7AAD). Results of Figure-1B depict that while cell-free MCF-7 supernatants caused apoptosis to human peripheral CD4^+^ T cells (38.4% in supernatant-treated cells and 5.5% in control) as well as Jurkat cells (42.5% in supernatant-treated cells and 8.2% in control), theaflavins furnished protection to them. Interestingly, theaflavins did not change CD4^+^ cell numbers significantly when applied directly to control CD4^+^ T cells (Fig. 1B).

### Tumor-shed PGE_2_ is responsible for CD4^+^ T cell apoptosis

**Figure 2:**
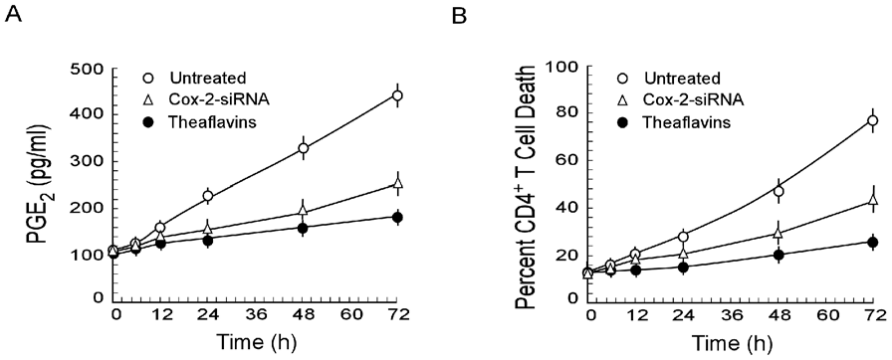
Tumor-shed PGE_2_ is responsible for CD4^+^ T cell apoptosis. A, Tumor-secreted PGE_2_ over time in cell-free spent media of MCF-7 cells (control (○), Cox-2-siRNA-transfected (Δ) or theaflavin-treated (•) was determined by ELISA. B, Percent CD4^+^ T cell death (Annexin-V-PE^+^/7AAD^+^), induced by the spent media as described in Fig. 2A, was plotted over time. Values are mean±S.E.M. of three independent sets of experiments.

All the reactions so far defined occurred independent of direct contact of CD4^+^ cells with tumor cells or even proximity thereby pointing towards the possibility of the presence of tumor-shed soluble immunosuppressors in the supernatant. Our search revealed a significant increase in PGE_2_ in tumor supernatant in a time-dependent manner with a maximum at 72 hours of continuous culture while media from theaflavins-treated MCF-7 cells contained significantly lower levels of this immunosuppressor (Fig. 2A). Moreover, percent CD4^+^ cell apoptosis was positively correlated (r = 0.964) with the PGE_2_ level of individual supernatant (Fig. 2B), whereas theaflavin-pre-treated tumor supernatants failed to induce significant death. Importantly, insertion of Cox-2-siRNA in tumor cells blocked the increase in PGE_2_ in cell-free tumor supernatant overtime (Fig. 2A). This suggested inhibition in the generation and/or release of PGE_2_ that resulted in lesser killing of CD4^+^ cells than untreated ones (Fig. 2B). All these data confirmed the role of Cox-2-derived PGE_2_ in tumor-induced CD4^+^ T cell killing. These results prompted us to explore the possibility of theaflavins to inhibit PGE_2_ production in tumor cells. Results of Figure 2 indeed depicted that theaflavin-treated tumor cells secreted significantly low level of PGE_2_ (Fig. 2A) that displayed lesser CD4^+^ cell killing (Fig. 2B)

### PGE_2_ perturbs IL2Rγ-signaling events in CD4^+^ T cells

**Figure 3:**
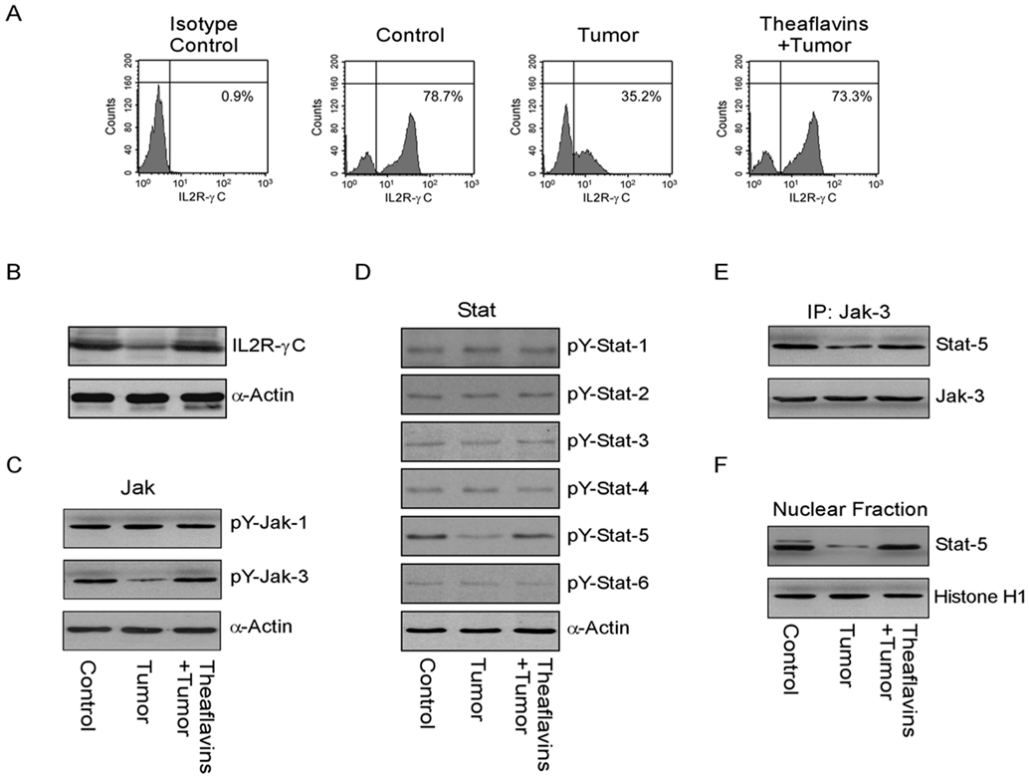
Tumor-PGE_2_ perturbs IL2Rγc signaling events in CD4^+^ T cells. Purified human CD4^+^ T cells, cultured in the presence of media alone or cell-free MCF-7-spent media (±theaflavins), were (A) subjected to flow cytometric analysis to determine the surface levels of IL2Rγc, or lysed for Western blotting of (B) IL2Rγc, (C) tyrosine phosphorylated Jak-1/Jak-3 and (D) tyrosine phosphorylated Stat-1/Stat-2/Stat-3/Stat-4/Stat-5/Stat-6 using specific antibodies. (E) Jak-3/Stat-5 complexes were immunopurified with anti-Jak-3 antibody from CD4^+^ T cell lysates. The immunopurified proteins were subjected to Western blot analysis to confirm association of Jak-3 with Stat-5. (F) Nuclear fractions from CD4^+^ T cells were Western blotted using anti-phospho-Stat-5 antibody to determine nuclear translocation of Stat-5. α-Actin and Histone H1 were used as internal loading controls for cytosol and nuclear fractions, respectively.

It is known that IL2 is essential for T cell homeostasis and activation for which it depends on IL2Rγc signaling [42], [43]. Thus, γc down regulation, if any by tumor-secreted PGE_2_, may fail to support T cells that then become susceptible to apoptotic death [43]. In our experimental model, we observed a marked decrease in the surface expression (Fig. 3A) as well as total amount (Fig. 3B) of IL2Rγ chain in CD4^+^ T cells that were co-incubated with tumor supernatants (Fig. 3A). Receptor expression in CD4^+^ T cells could be efficiently restored back to normal level by theaflavin-treatment of tumor cells (Fig. 3A and 3B).

It has been shown that Jak-3 is required to activate IL2R pathway for T cell proliferation [44] and loss of Jak-3 expression or kinase activity results in impaired activation of the IL2R signaling pathway [45]. Moreover, it is acknowledged that IL2Rγc is the primary mediator of cytokine signaling and activates Jak-3/Stat-5 signaling cascade [46] by Jak/Stat phosphorylation and subsequent translocation of phospho-Stat to nucleus [47]. We observed that, of the Jak proteins and associated Stat proteins, phosphorylations of Jak-3 and Stat-5 were down regulated in CD4^+^ T cells (Fig. 3B–C) by tumor-shed PGE_2_ (Fig. 2A), which could be ameliorated by pre-treatment of the tumor cells with theaflavins (Fig. 3B–C). To test whether Stat-5 was phosphorylated by Jak-3, we co-immunoprecipitated Stat-5 with anti-Jak-3 antibody, and the immunopurified proteins were then Western-blotted with anti-phospho-Stat-5 antibody. Results of Figure 3D showed that tumor supernatant substantially reduced association of phospho-Stat-5 with Jak-3 in CD4^+^ T cells. Perturbation in phospho-Stat-5 nuclear translocation activity was also observed in tumor-exposed CD4^+^ T cells in comparison to its untreated counterparts (Fig. 3E). Perturbation in phospho-Stat-5 nuclear translocation activity was also observed in tumor-exposed CD4^+^ T cells in comparison to its untreated counterparts (Fig. 3E). Interestingly, when CD4^+^ cells were cultured in presence of theaflavin-pretreated tumor cell supernatants, significant protection towards IL2Rγc/Jak-3/Stat-5 signaling i.e., up-regulation of γc expression, Jak-3/Stat-5 association and phosphorylation as well as nuclear localization of Stat-5 (Fig. 3B–E), was observed in CD4^+^ T cells, which offered them relive from the apoptotic insult that was observed with untreated tumor supernatant (Fig. 2B).

### Stat-5A transfection confers resistance to CD4^+^ T cells from tumor-induced death

**Figure 4:**
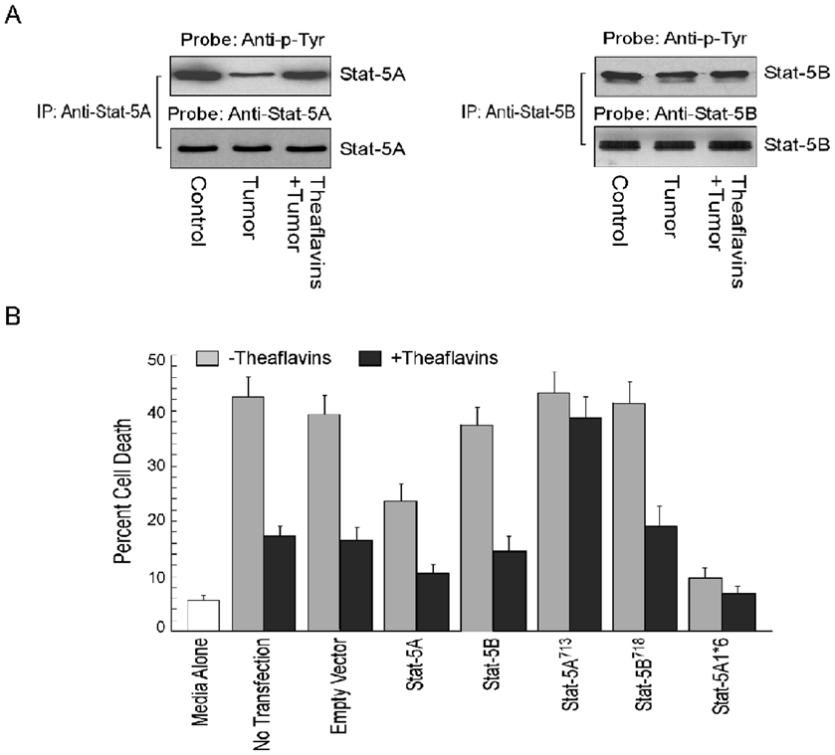
Stat-5A transfection confers resistance to CD4^+^ T cells from tumor-induced death. A, Stat-5A (*left panel*) and Stat-5B (*right panel*) isoforms were immunoprecipitated from cell lysates using specific antibodies and then Western blotted with anti-phospho-tyrosine or anti-Stat-5A/Stat-5B antibodies to determine phosphorylation status of specific proteins. B, Jurkat T cells were transfected with control vector, wild-type *Stat-5A/Stat-5B*, C-terminal truncated *Stat-5A*_713_/*Stat-5B*_718_ or constitutively active Stat-5A1*6 genes and were cultured in the presence of media alone or MCF-7 spent media (±theaflavins) for 48 h. Percent cell death (Annexin-V-PE^+^/7AAD^+^) was determined flow cytometrically. Values are mean±S.E.M. of three independent sets of experiments.

After confirming the Stat-5-mediated pathway as the major pathway in tumor-induced CD4^+^ T cell apoptosis, we undertook two different approaches to identify the isoform(s) of Stat-5 involved since both Stat-5A and Stat-5B isoforms play critical role in Bcl-2 induction in T cells. Results of Figure-4A depict that PGE_2_ present in cell-free tumor supernatant (Fig. 2A) significantly inhibited the phosphorylation of Stat-5A (Fig. 4A, *left panel*) but not that of Stat-5B (Fig. 4A, *right panel*). In this situation too, prior treatment of the tumor cells with theaflavins could bring back the phosphorylation status of Stat-5A to normal level in CD4^+^ T cells (Fig. 4A, *left panel*).

To further confirm the role of two Stat-5 isotypes in tumor-induced CD4^+^ T cell demise, Jurkat T cells were over-expressed with wild-type Stat-5A/Stat-5B, C-terminal truncated dominant negative *Stat-5* (*Stat-5A_713_* and *Stat-5B_718_*) or constitutively active *Stat-5A* (Stat-5A1*6) genes (Fig. 4B) and then incubated with tumor-culture supernatants. Multiple experiments demonstrated that unlike wild-type cells, constitutively active Stat-5A1*6-transfected cells were only minimally affected, with an average of only 6–10% of the transfected cells succumbing to a tumor-supernatant-induced apoptotic death and theaflavins were able to protect them further (Fig. 4B). On the other hand, when dominant negative *Stat-5A_713_* was introduced into Jurkat T cells, this protein rendered these cells more susceptible to tumor-induced death that could not be successfully prevented by theaflavin-administration. In contrast, theaflavins were able to protect *Stat-5B_718_*-transfected Jurkat T cells from tumor-induced death (Fig. 4B). When wild-type *Stat-5A* was introduced into these cells, it was able give protection from tumor-induced death, by itself or in the presence of theaflavins (Fig. 4B). All these results strongly reconfirm our hypothesis that Stat-5A protects CD4^+^ T cells from tumor-induced apoptosis and theaflavins utilize this isoform of Stat-5 to assert its protective effect.

### Tumor-shed PGE_2_ indulges a shift from pro-survival to pro-apoptotic environment in CD4^+^ T

**Figure 5:**
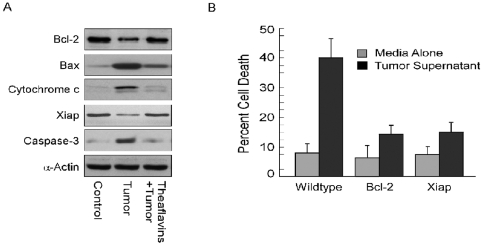
Tumor-shed PGE_2_ indulges a shift from pro-survival to pro-apoptotic environment in CD4^+^ T cells. A, Purified CD4^+^ T cells were cultured in the presence of media alone or MCF-7 spent media (±theaflavins) and cell lysates (for the determination of Bcl-2 and Bax) as well as cytosolic fractions (for cytochrome c release, Xiap expression and caspase-3 cleavage) were Western blotted using specific antibodies. α-Actin was used as internal loading control. B, Jurkat T cells, wild type as well as *Bcl-2* or *Xiap* transfected, were incubated with media alone or MCF-7-spent media for 48 h. Percent cell death was determined flow cytometrically. Values are mean±S.E.M. of four independent sets of experiments in each case.

It is acknowledged that the regulation of both pro- and anti-apoptotic Bcl-2 family proteins is dependent upon the IL2Rγ chain signals [43] involving Jak-3/Stat-5 cascade. Thus, γc down regulation and inhibition of Jak-3/Stat-5A signaling by PGE_2_ present in the tumor supernatant, as obtained here, may fail to support sustained Bcl-2 expression, leading to CD4^+^ T cells susceptibility towards apoptotic death. To verify this hypothesis, we examined the expression levels of anti-apoptotic Bcl-2 and pro-apoptotic Bax proteins in CD4^+^ T cells. Results of Figure-5A depict that tumor insult down-regulated Bcl-2 and up-regulated Bax thereby lowering the Bcl-2:Bax ratio and creating a pro-apoptotic environment in these helper cells. Moreover, the expression of Xiap (X-linked inhibitor of apoptosis protein), a member of the inhibitor of apoptosis family of proteins, was diminished in these T cells treated with tumor supernatant (Fig. 5A), making them more susceptible to execution by caspases, as Xiap is known to inhibit caspases-3, -7 and -9 [48].

To validate these results, Jurkat T cells were ectopically transfected with Bcl-2 construct. Multiple experiments demonstrated that although control T cells were highly sensitive to tumor supernatant, with an average of 42% of Jurkat cells testing positive for apoptosis, Bcl-2-transfected cells were only minimally affected, with an average of only 12% of the transfected cells succumbing to a tumor supernatant-induced apoptotic death (Fig. 5B). These results led us to ask the question whether over-expression of Xiap would also make these cells more resistant to tumor-induced death. In fact, when these cells were over-expressed with Xiap gene, less killing was observed (Fig. 5B).

It is appreciated that the increase in Bax may cause mitochondrial trans-membrane potential loss that results in the release of cytochrome c in CD4^+^ T cell cytosol leading to activation of caspase cascades and apoptosis [49]. The experiment depicted in Figure-5A clearly illustrates that culturing CD4^+^ T cells with MCF-7 cell free supernatant, a significant increase in cytochrome c level in cytosol was observed while in unexposed CD4^+^ T cell cytosol it was minimally detected (Fig. 5A). Infact, in the downstream, substantial activation of caspase-3 was observed in supernatant-treated CD4^+^ T cells. These results indicate a total impairment in IL2Rγc survival signaling in CD4^+^ T cells by tumor-secreted PGE_2._ All these factors were restored back to their original levels up on prior treatment of tumor cells with theaflavins (Fig. 5A) thereby protecting CD4^+^ cells from tumor-induced apoptosis.

### Re-confirmation of PGE_2_ as the molecule behind tumor-induced perturbation in CD4^+^ T cell survival signaling

**Figure 6:**
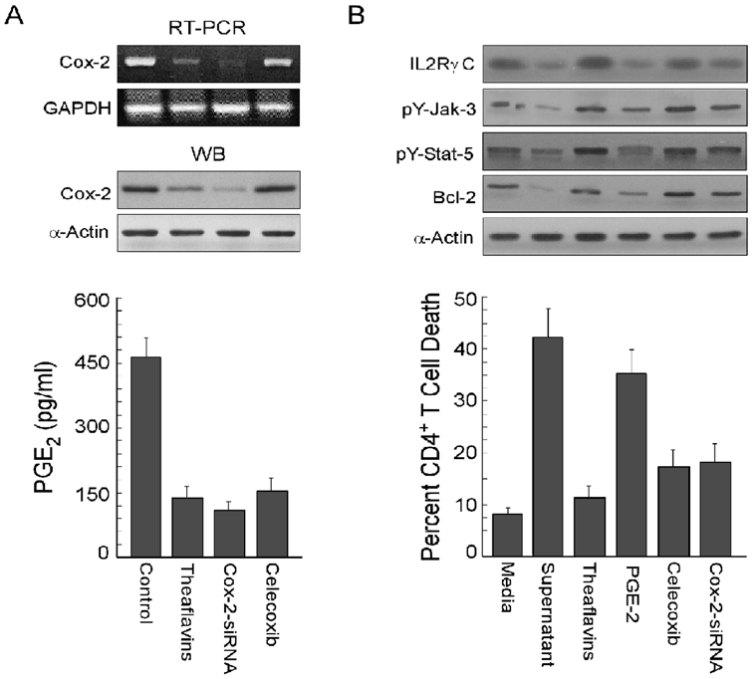
Re-confirmation of PGE_2_ as the molecule behind tumor-induced perturbation in CD4^+^ T cell survival signaling. A, MCF-7 cells were treated with theaflavins or celecoxib or transfected with Cox-2-siRNA and the levels of Cox-2 and GAPDH (internal control) mRNA were determined by RT-PCR (*upper panel*). Western blot analysis was performed for the determination of levels of Cox-2 or α-Actin (internal control) proteins (*middle panel*). In parallel experiments the amount of tumor-secreted PGE_2_ in the cell-free supernatant was determined by ELISA (*lower panel*). B, Purified CD4^+^ T cells were cultured in the presence of media alone or MCF-7-spent media (tumors were either pre-treated with 25 µg/ml theaflavins/3.5 ng/ml PGE_2_/50 µM celecoxib or transfected with 300pmole Cox-2-siRNA) for 48 h. Expression levels of IL2Rγc and Bcl-2 as well as phosphorylation status of Jak-3/-Stat-5 were determined by Western blotting in which α-Actin was used as internal control (*upper panel*). In parallel experiments, flowcytometric determination of percent cell death (*lower panel*) was established. Values are mean±S.E.M. of three independent experiments.

Finally, to re-confirm the role of tumor-shed PGE_2_ in materialising the CD4^+^ T cell killing effect of tumor, we undertook two approaches. In the first approach, when CD4^+^ T cells were cultured in presence of supernatants of tumor cells pre-treated with celecoxib or tranfected with Cox-2-siRNA. These treatments caused marked inhibition not only in Cox-2 expression (Fig. 6A, *upper panel*) but also in PGE_2_ production by the tumor cells (Fig. 6A, *lower panel*) and significant protection for the IL2Rγc signaling in CD4^+^ T cells i.e., up-regulation of γc expression, Jak-3/Stat-5 phosphorylation and increase in Bcl-2 level (Fig. 6B, *upper panel*), which offered these T cells relive from the apoptotic insult (Fig. 6B, *lower panel*). These findings were again validated in the second approach, in which treatment of CD4^+^ T cells with purified PGE_2_ down-regulated IL2Rγc survival signaling (Fig. 6B, *upper panel*) and induced apoptosis (Fig. 6B, *lower panel*). Results from these two approaches well confirmed the importance of tumor-shed PGE_2_ in impairment of IL2Rγc survival signal in CD4^+^ T cells that culminated to apoptosis. Above data also signify that normalization of this signaling pathway may be the mechanism by which theaflavins prevent tumor-induced caspase-3-dependent CD4^+^ T cell apoptosis.

## Discussion

A functional immune system is a potential barrier to tumor development and progression. To evade immune mechanisms, many tumors release immunosuppressive factors that attain high concentration *in situ* and inhibit cell-mediated effector function. Suppression of immune responses, coupled with superior growth kinetics of tumor cells, enable the neoplasm to surpass the control capacity of the host, leading to progressive decreases in cell-mediated anti-tumor responses and accelerated disease [50]. Although the immune system possesses the means to respond to cancer, it often fails to control the spread of malignancy. The fact that numerous laboratories, including our own, find that T lymphocytes undergo the physiological changes associated with apoptosis following co-culture with various cancer cell lines, lends support to the notion that it is the cancer cells themselves that induce lymphocyte death [14], [27]. Recent studies suggest that human carcinoma cells of various origins can activate apoptosis in lymphocytes interacting with the tumor *in vivo* and *in vitro* [51]–[53]. This tumor-induced apoptosis of lymphocytes may have important implications for the success of therapeutic regimens, including vaccination strategies [54].

Ample evidence suggests that CD4^+^ T cells facilitate the activation and development of anti-tumor responses of CD8^+^ T cells by enhancing clonal expansion at the tumor site, preventing activation-induced cell death and functioning as antigen-presenting cells for CTLs to preferentially generate immune memory cells [10]. CD4^+^ T cells assist CD8^+^ T cell priming *via* the engagement of CD40-ligand (CD154) on CD4^+^ T cells and CD40 expressed on DC [55]–[57]. This interaction results in the activation and maturation of DC, making them competent to stimulate antigen-specific CD8^+^ T cell responses [58]. It is important to note that primary CD8^+^ T cell responses to nonmicrobial antigens, as in case of cancer, display an absolute dependence on CD4^+^ T cell help [59], [60]. In this regard, our understanding of the importance of CD4^+^ T cells in orchestrating immune responses has grown dramatically over the past decade [10]. Here we aimed at elucidating the mechanisms of the tumor-induced suppression of such ‘T-cell help’ and its amelioration by theaflavins.

It has been demonstrated that several carcinoma cell lines produce soluble factors that inhibit T cell proliferation. Because tumor-induced apoptosis of lymphocytes may be mediated by an array of death receptors co-expressed on T cells or by tumor-derived soluble factors, it is important to characterize those intracellular events that may be potential targets for therapeutic intervention to minimize T cell apoptosis since if the immune system of cancer patient is persistently compromised, the success of any kind of therapy would be limited unless the immune system can be appropriately stimulated.

PGE_2_ has been implicated as a potential inhibitor of T cell function in the context of malignant disease [61], [62]. Elevated levels of PGE_2_ have also been found in patients with Hodgkin's lymphoma, which is suggested to be partially responsible for decreased cellular immune function in these patients [4], [5]. Another report confirmed that tumor over-expression of PGE_2_ *via* the elaboration of Cox-2 directly blocks patient's defence mechanism against cancer and promote cancer growth [63]. It is well known that PGE_2_ has diverse effects on CD4^+^ T cells leading to inhibition of T cell activation [64]. Albeit the outcome of PGE_2_-signaling is well established, the molecular mechanisms involved are still not completely understood. The present study was designed to determine the potential mechanisms of tumor-derived PGE_2_ leading to CD4^+^ T cell apoptosis. We exclusively used primary human CD4^+^ T cells to achieve more physiologic conditions compared with cell line models defective in key enzymes of T cell signaling [65]. Our study demonstrated the presence of Cox-2 derived PGE_2_ in tumor supernatant and that the effect of tumor supernatant on CD4^+^ T cells bore remarkable similarities with the effects of PGE_2_ exposure on these cells. Analysis of data revealed that PGE_2_ present in cell free tumor supernatant interferes with one of the major pathways for survival and activation of CD4^+^ T cells. This interference affected γc expression, Jak-3/Stat-5 phosphorylation and Bcl-2 level suggesting an inhibition of early IL2Rγc-mediated signaling events in CD4^+^ cells. We showed that PGE_2_ leads to down-regulation of IL2Rγc expression and Jak-3 phosphorylation thereby inactivating the most proximal events of IL2Rγc signaling. Inability of externally added IL2 to overcome tumor-PGE_2_ effect indicated that IL2 deprivation is not the primary cause of CD4^+^ cell death in our system.

Alternatively, theaflavins, which inhibited Cox-2 expression and subsequent PGE_2_ production in tumor cells, efficiently ameliorated tumor-induced impairment of IL2Rγc signaling. Recovery of Jak/Stat signaling also altered Bcl-2:Bax ratio in these helper cells in favour of survival. These results are in contrary to those of Kolenko *et al.* [26] who showed that in renal cancer, impairment in IL2R signaling by PGE_2_ did not affect IL2-dependent induction of Bcl-2. However, studies with Jak-3^−/−^ knockout mice showing impaired T cell development and severe down-regulation of Bcl-2 protein in T cell populations with moderate increase in Bax expression [66], [67] strengthened our findings. Interestingly, theaflavins offered better protection to T helper cells from tumor-induced death in comparison to either celecoxib or Cox-2-siRNA suggesting that along with PGE_2_, tumor-induced CD4^+^ T cell death might have other mediators that are independent of Cox-2 and inhibition of not only PGE_2_ but also those mediators by theaflavins could be the cause behind observed immunoprotection provided by these polyphenols.

In conclusion, our data provide direct evidence that tumor-derived Cox-2-dependent PGE_2_ affects IL2Rγc-driven signals in CD4^+^ T cells by down-regulating IL2Rγc and inactivating Jak resulting in impaired activation of downstream signals as shown by decreased phosphorylation of Stat. As a consequence, Stat phosphorylation and translocation to nucleus as well as anti-apoptotic Bcl-2 and Xiap protein expressions were decreased while increasing Bax thereby activating mitochondrial death cascade in these cells. The functional consequence is a state of CD4^+^ T cell depletion. Although CD4^+^ T cells are central to the function of the immune system, their role in tumor immunity remains under-appreciated. Recent advances in understanding of contribution of CD4^+^ in anti-tumor immunity, as well as some intriguing developments in the clinic, indicate that due to the cooperative role of CD4^+^ helper T cells for CD8^+^ cytotoxic T cells, they have the unrealized potential in tumor eradication. Based on our observations, we postulate PGE_2_ as an important factor responsible for impaired survival signaling in CD4^+^ T cells thereby leading them towards apoptosis in tumors associated with higher PGE2 production. In addition, our results demonstrating theaflavin-induced protection of IL2Rγc/Jak-3/Stat-5A signaling as well as survival of CD4^+^ T cells from tumor PGE_2_-insult signify that these plant polyphenols may have the potency to sustain the cell mediated immunity of the cancer-bearer that can be utilized for developing an effective therapy of this deadly disease.

## Materials and Methods

### Isolation of CD4^+^ T cells

Human venous blood from healthy adult volunteers was collected with informed consent using heparinized syringes. Whole blood (100 mL) was diluted with 150 mL of RPMI 1640 (Sigma, St Louis, MO, USA) and then layered in centrifuge tubes onto 120 mL of Histopaque-1077 (Sigma) gradient. After centrifugation the opaque interface containing lymphocytes was collected, washed twice in RPMI 1640 and, after complete supernatant removal, the pellet was re-suspended in PBS supplemented with 0.5% of BSA and 2 mmol/L EDTA. CD4^+^ T cells were purified from total leukocytes by positive selection using anti-CD4 antibody coated micro-beads (Milteny Biotech) [68]. The purity of the isolated CD4^+^ T cells was determined by flow cytometry and was routinely >99% CD3^+^ and CD4^+^, but was negative for CD8. Cells were cultured in RPMI 1640 (supplemented with 10 U/ml recombinant IL-2, 10% fetal bovine serum, 2 mM L-glutamine, 100 µg/ml sodium pyruvate, 100 µM non-essential amino acids, 100 µg/ml streptomycin and 50 U/ml penicillin; Sigma) at 37°C in humidified incubator containing 5% CO_2_. Viable cell numbers were determined by Trypan blue exclusion test. The Jurkat T cell line (maintained in complete RPMI 1640) and human mammary epithelial carcinoma cells (MCF-7, MDA-MB-231 and ZR-75-1; maintained in complete DMEM) were obtained from NCCS, India. Tumor supernatants freed from cellular components were used in 1∶1 ratio with RPMI to study the effect of tumor supernatant on CD4^+^ T cells.

Tumor cells were pre-treated with different doses of theaflavins (6.25–50 µg/ml), as per the requirement of the experiment, for 90 min after which the culture media containing extra theaflavins were replaced by fresh media. Seventy two-hour old cell-free tumor supernatants were used in 1∶1 ratio with RPMI for 48 h to study the effect of tumor on CD4^+^ T/Jurkat cells. To study the role of tumor-shed PGE_2_ on CD4^+^ T apoptosis, supernatants of Cox-2 inhibitor-treated (celecoxib; 50 µM) or Cox-2-siRNA-transfected tumor cells were used. In parallel sets of experiments CD4^+^ T cells were incubated with 3.5 ng/ml of prostaglandin E_2_ (Sigma).

### Plasmid constructs, siRNA and transfections

Flag-tagged (N-terminus) cDNA encoding full length Stat-5A and Stat-5B, C-terminal truncated Stat-5A (amino acid 713; Stat-5A_713_) and Stat-5B (amino acids 718; Stat-5B_718_) (provided by Dr. J.N. Ihle of St. Jude Children's Research Hospital, TN), constitutively active mutant Stat-5A1*6 (generous gift from Prof. T. Kitamura, Institute of Medical Science, University of Tokyo, Japan), Bcl-2, Xiap in Prk5 vector (2 µg/million cells) were introduced separately into Jurkat T cell using T cell nucleofector kit (Amaxa, Koein, Germany). Isolation of stably expressing clones were done by limiting dilution and selection with IL2 (25 U/ml) and hygromycin B (800 mg/ml) for 14 days and cells surviving this treatment were cloned and assessed for Bcl-2 and Stat-5 expressions by Western blot analysis. Tumor cells were transfected with 300pmole of Cox-2-/control-ds-siRNA (Santa Cruz) and lipofectamine 2000 separately for 12 h. Levels of Cox-2 mRNA and protein were estimated by RT-PCR and Western blotting. Transfected cells were cultured for 72 h and cell free supernatants were collected for subsequent experiments.

### Flow cytometry

For the determination of cell death, CD4^+^ T cells were labelled with 7-aminoactinomycin D (7-AAD) and Annexin-V-phycoerythrin (PE) (BD Bioscience) and analyzed on flow cytometer (FACS Calibur; Becton Dickinson), equipped with 488 nm Argon laser light source using CellQuest software. Electronic compensation of the instrument was done to exclude overlapping of the emission spectra. Total 10,000 events were acquired and cells were properly gated for analysis. Annexin-V^+^/7-AAD^+^/CD4^+^ cells were regarded as apoptotic cells. To determine the surface levels of IL2Rγc expression, CD4^+^ T cells were incubated with anti-IL2Rγc (2 µg/ml; Santa Cruz) antibody and then with FITC-conjugated 2nd antibody followed by flowcytometric analysis.

### Co-immunoprecipitation and immunoblotting

Primary human CD4^+^ T cells were lysed in buffer (10 mM Hepes, pH 7.9, 1.5 mM MgCl_2_, 10 mM KCl, and 0.5 mM DTT) and nuclei were pelleted by brief-centrifugation. The supernatant was spun at 105,000×g to get cytosolic fraction. The nuclear extract was prepared in buffer containing 20 mM HEPES, pH 7.9, 25% (v/v) glycerol, 420 mM KCl, 1.5 mM MgCl_2_, 0.2 mM EDTA, 0.5 mM DTT, and 0.5 mM PMSF. For whole cell lysate, cells were homogenized in buffer (20 mM Hepes, pH 7.5, 10 mM KCl, 1.5 mM MgCl_2_, 1 mM Na-EDTA and 1 mM DTT). All the buffers were supplemented with protease and phosphatase inhibitor cocktails [69], [70]. For direct Western blot analysis, cell lysates or the particular fractions containing 50 µg protein was separated by SDS-polyacrylamide gel electrophoresis and transferred to nitrocellulose membrane. The protein of interest was visualized by chemiluminescence.

For the determination of direct interaction between two proteins, co-immunoprecipitation technique was employed. The immunopurified proteins were then detected by Western blot using specific antibody (Santa Cruz). Equal protein loading was confirmed by re-probing the blots with α-actin/histone H1 antibody (Santa Cruz).

### Prostaglandin E_2_ assay

PGE_2_ content in culture supernatants was determined using PGE_2_ ELIZA bioassay kit (US Biologicals) following the manufacturer's protocol.

### Statistical Analysis

Values are shown as standard error of mean (SEM) except otherwise indicated. Comparison of multiple experimental groups was performed by 2-way ANOVA followed by a post-hoc Bonferroni multiple comparison test. Data were analyzed and, when appropriate, significance of the differences between mean values was determined by a Student's t test. Results were considered significant at p<0.05.
